# Trop-2 as an Actionable Biomarker in Breast Cancer

**DOI:** 10.2174/1389202924666230726112233

**Published:** 2023-11-22

**Authors:** Giulia Cursano, Emanuele Frigo, Elham Sajjadi, Mariia Ivanova, Konstantinos Venetis, Elena Guerini-Rocco, Carmen Criscitiello, Giuseppe Curigliano, Nicola Fusco

**Affiliations:** 1 Division of Pathology, IEO, European Institute of Oncology IRCCS, Milan, Italy;; 2 School of Pathology, University of Milan, Milan, Italy;; 3 Department of Oncology and Hemato-oncology, University of Milan, Milan, Italy;; 4 Division of New Drugs and Early Drug Development for Innovative Therapies, IEO, European Institute of Oncology IRCCS, Milan, Italy

Human trophoblastic cell surface antigen 2 Trop-2 is a calcium signal transducer originally identified in trophoblast cells but subsequently found to be highly expressed in various solid cancers, including breast cancer [1, 2]. It is encoded by *TACSTD2*, which is located on chromosome 1p32 [3]. Expression of this intronless gene was observed in all breast cancer subtypes, specifically in luminal A and triple-negative [4, 5]. From the biological standpoint, upregulation of Trop-2 is associated with accelerated tumor growth and unfavorable prognosis, being involved in several cancer-related pathways *e.g*., PI3K/AKT/mTOR, JAK/STAT, MAPK/ERK, Wnt/β-catenin [4]. A new class of antibody-drug conjugates (ADCs) has been developed to deliver activity to both Trop-2 expressing cells and the tumor microenvironment [6]. Among these, Sacituzumab govitecan (SG) was the first Trop-2 directed ADC approved by the FDA in April 2020 for patients with pre-treated metastatic breast cancer, either of triple-negative or hormone receptor (HR)-positive, HER2-negative IHC score 0-2+/ISH- phenotypes [7]. The FDA approval was based on significant progression-free survival and overall survival data from the phase 3 ASCENT NCT02574455 and TROPiCS-02 NCT03901339 studies [8]. Furthermore, a phase I/II study has demonstrated the efficacy of single-agent SG also in the neoadjuvant setting [9]. In April 2021, the FDA granted another accelerated approval to Sacituzumab govitecan for the treatment of advanced urothelial cancer. This approval was primarily supported by data from cohort 1 of the TROPHY-U-01 phase II study [10]. Currently, the recruiting phase 1 TROPION-PanTUMOR01 and phase 3 TROPION-Breast02 studies are investigating a new Trop-2 directed ADC, Datopotamab Deruxtecan Dato-Dxd. Preliminary results have shown promising antitumor activity of Dato-DXd in advanced triple-negative breast cancer (TNBC) [11].

Due to its ubiquitous expression in cancer cells with a relatively low expression in non-neoplastic epithelial cells, as well as its critical role in the metastasis and progression of many cancers, Trop-2 emerges as a trustworthy candidate biomarker [12]. This protein has been previously investigated in thyroid pathology research, with significant results as a diagnostic biomarker for papillary thyroid carcinoma [13]. In this study, the authors used an immunohistochemical (IHC) assay (F-5 clone, sc-376181, Santa Cruz Biotechnology, Inc) to identify and quantify Trop-2 expression, considering positive membranous staining in >5% of tumor cells [14]. Interestingly, a retrospective analysis of the ASCENT study revealed Trop-2 overexpression in 62% (290/468) of breast cancers [15]. For this analysis, the investigators employed a mouse monoclonal antibody for IHC and analyzed the expression using the H-score. Significant treatment benefits were observed in tumors with high/medium levels of Trop-2 expression compared with standard-of-care chemotherapy. However, the relatively small number of patients with low-to-negative Trop-2 precluded any further conclusion [15]. Earlier basket phase 1-2 studies, including breast cancer patients, used a goat polyclonal anti-Trop-2 antibody (R&D Systems), detected by a VECTASTAIN ABC kit with a 10% cut-off cell staining considered as positive [16]. This study reported positivity rates ranging from 73% to 85%. However, one study using a 10% positivity threshold found a lower TROP2 protein expression rate in TNBC (56%). Moreover, significant discrepancies in TROP2 expression were observed between primary and metastatic TNBC, highlighting intratumoral heterogeneity [17]. In a phase I study PF-06664178, an anti-Trop-2/Aur0101 ADC in patients with advanced or metastatic solid tumors, a specific IHC assay was developed in-house and optimized by identifying four control lines by flow cytometry, Western Blot, qRT-PCR, and Affymetrix chips [18]. Western blot provided protein abundance and molecular weight information, qRT-PCR quantified mRNA levels, and Affymetrix chips enabled high-throughput gene expression analysis. These techniques collectively yielded a comprehensive understanding of TROP2 expression and its implications as a therapeutic target or biomarker in solid tumors. Although overexpression of Trop-2 was not a requirement for study participation, no correlation was found between Trop-2 expression and the objective response. In breast cancer, Trop-2 has been tested using an Anti-Trop2 B9 sc-376746 antibody (Santa Cruz), scoring the staining intensity from 0 to 3 [19]. Another assay previously used in TNBC translational research is the Ab227689 (Abcam) on the Bond Automatic IHC Stainer (Leica Biosystems) [20]. In this study, the authors found an association between Trop-2 and apocrine phenotype [20]. A summary of clinical trials enrolling patients with breast cancer in which Trop-2 status is being tested, along with the specific methodology, is provided in Table **1**.

The expression of Trop-2 in breast cancer cells is associated with poor prognosis and increased risk of metastasis [3]. With the development of the new anti-Trop2 ADCs in breast cancer, there is now room for the implementation of tailored Trop-2 testing strategies in pathology to improve treatment plans. In addition, Trop-2 testing can help guide the selection of treatment options for patients who are resistant to traditional therapies. This type of analysis is likely to play a growing role in the management and treatment of breast cancer patients, providing the realization of standardized and harmonized testing methods. It is also important to consider the heterogeneity in Trop-2 expression and the clinical role between TNBC and HR-positive diseases. In conclusion, despite the absence of companion diagnostics in the table.

In the Trop-2 prospective studies, the development of a reliable testing strategy would be of clinical value in breast cancer, both for patients’ selection and for clinical trial design.

## Figures and Tables

**Table 1:** Overview of clinical trials assessing Trop-2 expression in breast cancer with the corresponding methodology for testing.

**Author**	**NCT**	**Study Phase**	**Drug**	**Method**	**Assay**	**Platform**	**IHC Analysis**	**Tumor Type**
Starodub *et al*. 2015	01631552	Phase I/II IMMU-132-01	Sacituzumab govitecan	IHC (FFPE), ELISA (serum)	Goat polyclonal antibody anti-human Trop-2 (R&D Systems)	Dako	Score 0 to 3+0: ≤10% 1+: >10% weak 2+: >10% moderate 3+: >10% intense	Solid tumors
Bardia *et al*. 2021	02574455	Phase III ASCENT	Sacituzumab govitecan	IHC (FFPE)	Mouse monoclonal antibody anti Trop-2 (ENZ ABS 380-0100, Enzo Life Science, Farmingdale, NY)	Ventana	H-Score 0-100: low; 100-200: intermediate; >200: high	TNBC
King *et al*. 2018	02122146	Phase I	PF-06664178	IHC, WB, qRT-PCR, Affymetrix chips (FFPE)	In-house developed assay	Dako	Score 0 to 3+ Moderate (>50% of 2+ cells) High (>50% of 3+ cells)	Solid tumors

**Abbreviations:** IHC, immunohistochemistry; FFPE, formalin-fixed paraffin-embedded; TNBC, triple-negative breast cancer.
